# An Ultra-High-Q Lithium Niobate Microresonator Integrated with a Silicon Nitride Waveguide in the Vertical Configuration for Evanescent Light Coupling

**DOI:** 10.3390/mi12030235

**Published:** 2021-02-25

**Authors:** Jianhao Zhang, Rongbo Wu, Min Wang, Youting Liang, Junxia Zhou, Miao Wu, Zhiwei Fang, Wei Chu, Ya Cheng

**Affiliations:** 1 State Key Laboratory of High Field Laser Physics and CAS Center for Excellence in Ultra-Intense Laser Science, Shanghai Institute of Optics and Fine Mechanics, Chinese Academy of Sciences, Shanghai 201800, China; jhzhang@siom.ac.cn (J.Z.); rbwu@siom.ac.cn (R.W.); 2 Center of Materials Science and Optoelectronics Engineering, University of Chinese Academy of Sciences, Beijing 100049, China; 3 State Key Laboratory of Precision Spectroscopy, East China Normal University, Shanghai 200241, China; 15253172638@163.com (Y.L.); 52180920026@stu.ecnu.edu.cn (J.Z.); wumiao1993@126.com (M.W.); zwfang@phy.ecnu.edu.cn (Z.F.); 4 The Extreme Optoelectromechanics Laboratory (XXL), School of Physics and Electronic Science, East China Normal University, Shanghai 200241, China; 5 Collaborative Innovation Center of Extreme Optics, Shanxi University, Taiyuan 030006, China; 6 Collaborative Innovation Center of Light Manipulations and Applications, Shandong Normal University, Jinan 250358, China; 7 CAS Center for Excellence in Ultra-Intense Laser Science, Shanghai 201800, China; 8 Shanghai Research Center for Quantum Sciences, Shanghai 201315, China

**Keywords:** lithium niobate microring resonator, silicon nitride waveguide, photolithography assisted chemo-mechanical etching

## Abstract

We demonstrate the hybrid integration of a lithium niobate microring resonator with a silicon nitride waveguide in the vertical configuration to achieve efficient light coupling. The microring resonator is fabricated on a lithium niobate on insulator (LNOI) substrate using photolithography assisted chemo-mechanical etching (PLACE). A fused silica cladding layer is deposited on the LNOI ring resonator. The silicon nitride waveguide is further produced on the fused silica cladding layer by first fabricating a trench in the fused silica while using focused ion beam (FIB) etching for facilitating the evanescent coupling, followed by the formation of the silicon nitride waveguide on the bottom of the trench. The FIB etching ensures the required high positioning accuracy between the waveguide and ring resonator. We achieve Q-factors as high as 1.4 × 10^7^ with the vertically integrated device.

## 1. Introduction

Lithium niobate (LN) has long been recognized as an important material platform for integrated photonic devices because of its wide transparent window, high nonlinear coefficient, and excellent electro-optical property [1]. In particular, the latest advancement of the fabrication technologies of high-quality photonic micro- and nanostructures on lithium niobate on insulator (LNOI) has further promoted the development of integrated photonics on the LNOI platform. The building block photonic structures, such as microresonator and optical waveguide, are typically fabricated on LNOI substrate using either maskless focused ion beam (FIB) milling or lithographic processing involving argon ion milling [2,3,4,5,6,7,8,9,10,11]. Meanwhile, a chemo-mechanical etching process (termed photolithography assisted chemo-mechanical etching (PLACE) hereafter) has been developed to achieve ultra-high surface smoothness, resulting in high-quality (high-Q) microdisk resonator with a Q-factor of 4 × 10^7^ and ultra-low-loss optical waveguide with a propagation loss of 0.03 dB/cm [12,13,14]. So far, a broad range of nonlinear optical processes have been demonstrated with the ultra-high-Q LN microresonators that were fabricated using the PLACE technology, ranging from optomechanics [15] and optical frequency comb [16] to nonlinear frequency conversion [17] and on-chip micro-disk lasing.

Light must be efficiently coupled into the microresonaotors using either a fiber taper [18] or an integrated optical waveguide [19] in order to excite the nonlinear optical effects. The on-chip integration of the microresonator and the coupling optical waveguide provides an efficient means for up scaling of the photonic integration circuits (PICs), which is critical for some applications, such as photonic computation and quantum information processing [20], etc. However, in the PLACE scheme, the low-loss optical waveguides and high Q microresonators are both generated using the chemical-mechanical polishing (CMP) technique. In the CMP process, it is required that the distance between the closely located photonic structures should be on the micrometer scale, but not the nanometer scale; otherwise, the LNOI in the narrow gap between the neighboring structures cannot be efficiently removed by polishing. In this case, the lateral evanescent coupling between a microresonator and a waveguide is difficult to achieve, simply because of the fact that the evanescent coupling in the visible and near infrared ranges requires the gap width to be in the order of a few hundred nanometers. For this reason, on-chip evanescent coupling has not been realized between an optical waveguide and a microresonator fabricated using the PLACE technique.

Here, we overcome the difficulty by utilizing a vertical coupling scheme between a crystalline LN microring resonators and a silicon nitride (Si_3_N_4_) waveguide. Si_3_N_4_ is also considered to be an attractive candidate for monolithic integration of photonic circuits because of its low propagation loss. Importantly, Si_3_N_4_ has a refractive index that is close to that of LN, which makes it easy to fulfill the phase matching condition between the Si_3_N_4_ waveguide and LN microresonator. We characterized the integrated device by measuring the Q-factor of the fabricated LN microresonator, and demonstrated the coupling control by varying the thickness of the SiO_2_ cladding layer.

## 2. Materials and Methods

**Figure 1:**
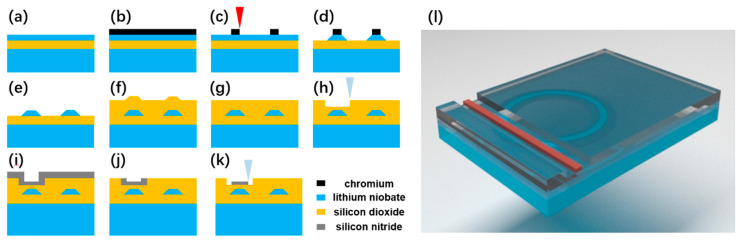
(**a**–**k**) Process flow of fabricating a lithium niobate (LN) microring resonator coupling with a silicon nitride (Si_3_N_4_) waveguide in the vertical configuration. (**l**) Three-dimensional (3D) diagram of the coupling structure.

**Figure 2:**
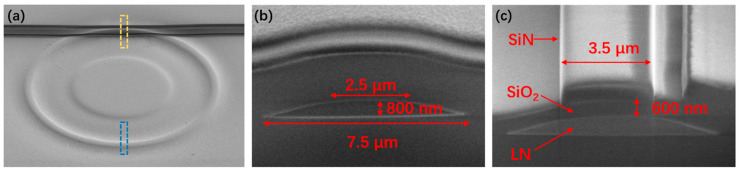
(**a**) Top view scanning electron micrograph (SEM) image of LN microring resonator and Si_3_N_4_ waveguide. (**b**) Sectional view SEM image of the structure at the location of the bule dashed box in (**a**). (**c**) Sectional view SEM image of the structure at the location of the yellow dashed box in (**a**).

The microring resonator is fabricated on a commercially available x-cut LNOI wafer with a thickness of 900 nm (NANOLN, Jinan Jingzheng Electronics Co., Ltd., Jinan, Shandong, China). The LN thin film is bonded to a 2 μm-thick SiO_2_ layer supported by a 500-μm-thick LN substrate. Figure 1a depicts the configuration of the LNOI wafer, followed by the schematic of process flow, as shown in Figure 1b–k. In general, the fabrication procedures include: (1) the deposition of a thin layer of chromium (Cr) with a thickness of 400 nm on the surface of the LNOI by magnetron sputtering (Figure 1b); (2) space-selective ablation of a Cr layer coated on top of the LNOI to generate the pattern of the microring resonator using a focused femtosecond laser beam (Figure 1c). In this step, the femtosecond laser ablation was conducted by a commercial laser system (Pharos, LightConversion, Lithuania) at a repetition rate of 500 kHz and a scan speed of 40 mm/s. The center wavelength of the femtosecond laser was 1030 nm, and the pulse width was set to be ~270 fs. A 100× objective lens (M Plan Apo NIR, Mitutoyo, Japan) with a numerical aperture (NA) of 0.7 was employed to pattern the Cr layer in order to obtain a high ablation resolution. Femtosecond laser ablation was carried out by translating the sample with a three-dimensional (3D) motion stage (ABL1500-ANT130, Aerotech Inc., USA); (3) etching of the LNOI layer by CMP (Figure 1d). In this step, the LN without being covered by the Cr mask will be completely removed, while the LN protected by Cr mask will survive from the CMP because of the high hardness of Cr; (4) removal of the residual Cr mask left on the surface of LNOI by chemical wet etching, and further eliminate the roughness by a second CMP process (Figure 1e); (5) the deposition of the SiO_2_ film on the LNOI waveguide to form the cladding layer by plasma enhanced chemical vapor deposition (PECVD) (Figure 1f); (6) polishing the surface of SiO_2_ cladding layer with the third CMP (Figure 1g); (7) patterning of the SiO_2_ layer while using focused ion beam (FIB) etching (Figure 1h). In particular, the depth of the etched trench can be controlled with an accuracy of ~1 nm using the FIB etching; (8) the deposition of a Si_3_N_4_ film on the SiO_2_ layer by PECVD to fill the trench fabricated in the SiO_2_ cladding layer (Figure 1i); (9) removing the Si_3_N_4_ above the SiO_2_ layer with the fourth CMP (Figure 1j); and, (10) patterning of the Si_3_N_4_ film in the trench using FIB etching to form the waveguide (Figure 1k). More details of the femtosecond laser micromachining of Cr, the CMP processing, and the FIB etching can be found elsewhere [12,13,14]. Figure 1l shows a schematic 3D view of the hybrid LN and Si_3_N_4_ coupling structure.

## 3. Results

Figure 2a shows the top-view scanning electron micrograph (SEM) of the vertically coupled LN microring and Si_3_N_4_ waveguide. The profiles of the microring buried beneath SiO_2_ layer can be distinguished in the SEM image. The radius of the LN microring is 50 μm. The cross section of the LN microring, as indicated by the blue dashed area in Figure 2a, is shown in Figure 2b. In the current design, the LN microring has a trapezoidal cross-section with a top width of 2.5 μm, a bottom width of 7.5 μm, and a height of 800 nm, which is covered by a 1.5-μm-thick SiO_2_ cladding layer. The cross section of the coupling area (yellow dashed area in Figure 2a) is shown in Figure 2c. The three-layer structure, i.e., LN, SiO_2_ and Si_3_N_4_ from bottom to top, can be clearly seen. The thickness of the PECVD SiO_2_ layer was reduced from 1.5 μm to 600 nm by FIB etching for evanescent light coupling. The rib Si_3_N_4_ waveguide was fabricated by FIB etching with a width of 3.5 µm and a rib height of 550 nm.

**Figure 3:**
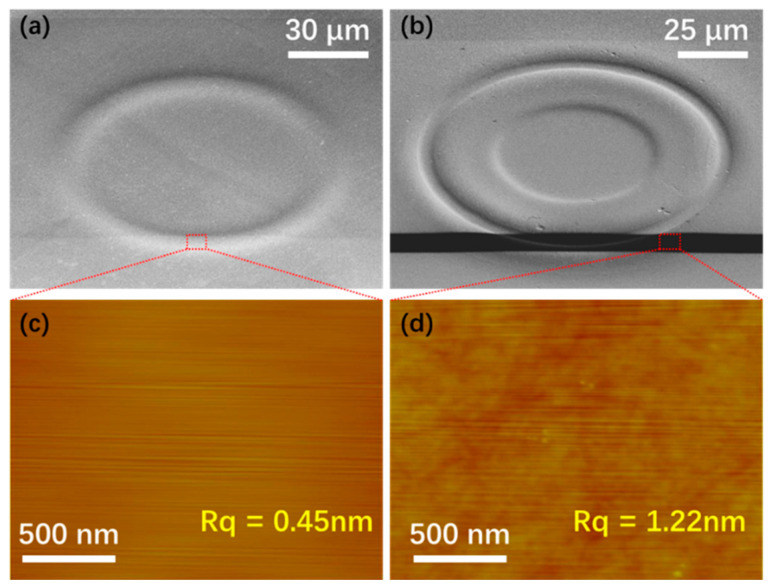
(**a**) Top view SEM image of the fabricated LN microring resonator. (**b**) Top view SEM image of the trench fabricated in the SiO_2_ cladding layer. (**c**,**d**) Atomic force microscope (AFM) images of the surfaces of the microring in (**a**) and the trench in (**b**) respectively.

Furthermore, we measured the surface roughness for both the LN microring resonator fabricated by CMP and the trench in SiO_2_ layer (i.e., the bottom of the trench) generated by FIB etching that are critical in obtaining high Q factors. Figure 3a,b present the SEM images of fabricated LN microring after CMP (step e in Figure 1) and the etched trench of the SiO_2_ layer using FIB etching (step h in Figure 1). Using an atomic force microscope (AFM), we measured the surface root-mean-square roughness (Rq) in the areas that are indicated by the red squares in Figure 3a,b respectively. An ultralow surface roughness of Rq ~ 0.45 nm can be achieved by the CMP processing, while a slightly higher roughness of Rq ~ 1.22 nm was achieved after FIB etching. Based on the smooth surface morphology, we can expect a high-Q LN microring resonator.

**Figure 4:**
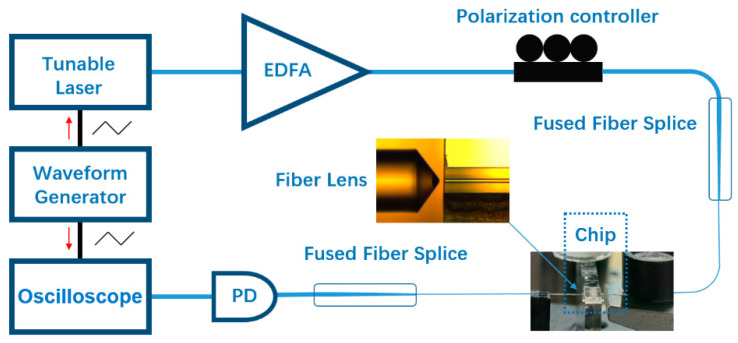
Schematic of experimental testing setup. Inset: optical micrograph of the fiber lens coupling with the Si_3_N_4_ waveguide.

We used an experimental setup to examine the coupling effect of the configuration and characterize the optical mode structure of the LN ring resonator, as schematically shown in Figure 4. A tunable laser (TLB 6728, New Focus Inc., San Jose, CA, USA) was employed to couple light into and out of Si_3_N_4_ waveguide through the lensed fiber with a taper angle of 90°. In order to enhance the detection signal, the tunable laser was boosted by an erbium-ytterbium-doped fiber amplifier (EYDFA, Golight, Inc., Culbertson, NE, USA) before coupling into the Si_3_N_4_ waveguide. The linewidth of the tunable laser is 200 kHz. The polarization of the pump laser was adjusted by an in-line fiber polarization controller. A photodetector measured the transmission of resonant mode (New focus 1811-FC-AC, Newport Inc., Irvine, CA, USA). We used an arbitrary waveform generator (AFG3052C, Tektronix Inc., Beaverton, DC, USA) to synchronize the tunable laser and oscilloscope signals.

**Figure 5:**
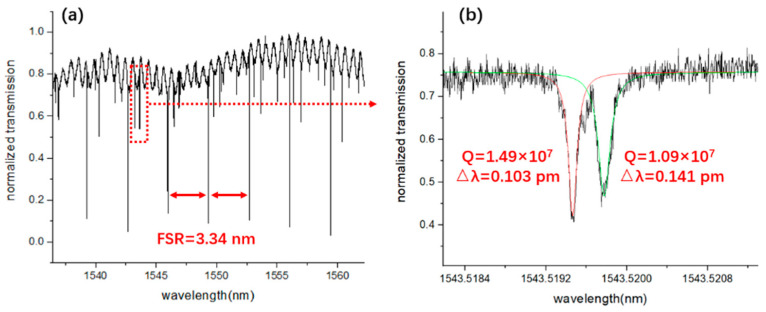
(**a**) Transmission spectrum of the LN microring resonator. (**b**) The Lorentz fitting of the splitting modes at the location of the red dotted box in Figure 4a reveals a Q-factor of 1.49 × 10^7^ (red solid line) and 1.09 × 10^7^ (green solid line), respectively.

Figure 5a shows the transmission spectrum for the wavelength range from 1537 nm to 1562 nm. The free spectral range (FSR) of the microresonator was measured to be 3.34 nm. A pair of the splitting whispering-gallery modes at the resonant wavelength around 1543.52 nm was chosen for the measurement of the Q-factor by fitting with a Lorentz function. The Q factors were measured to be 1.49 × 10^7^ and 1.09 × 10^7^, respectively, as indicated by the Lorentz curves shown in Figure 5b. The Q factors vary from 2.9 × 10^6^ to 1.49 × 10^7^, which is mainly caused by the different coupling condition of the whispering gallery modes. The high Q-factor of the LN microresonator indicates that the fabricated device with the vertical integration configuration functions effectively for evanescent light coupling between the LN microring and the Si_3_N_4_ waveguide.

**Figure 6:**
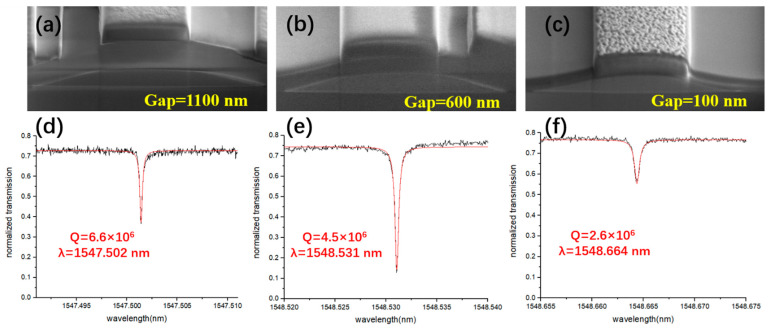
Sectional view SEM images of the coupling structures when the thickness of the SiO_2_ cladding layer are 1100 nm (**a**), 600 nm (**b**), and 100 nm (**c**), respectively. The Lorentz fitting of the modes of the structures in (**a**–**c**) are illustrated in (**d**–**f**), respectively.

Finally, we demonstrated that, in our scheme, the coupling efficiency as well as Q-factor can be tuned by changing the thickness of the SiO_2_ cladding layer, i.e., the vertical distance between the Si_3_N_4_ waveguide and the LN microring. The distance was adjusted by precisely controlling the FIB etching depth of the PECVD SiO_2_ layer. When the thickness of the SiO_2_ cladding layer was set to 1100 nm, the coupling efficiency was relatively low, leading to the undercoupling between the Si_3_N_4_ waveguide and the LN microring and giving rise to a high Q-factor of 6.6 × 10^6^, as illustrated in Figure 6a,d. Subsequently, we adjusted the distance to 600 nm, as shown in Figure 6b. The transmission loss of the light in the microring was close to the coupling loss between the waveguide and microring, which indicated that the critical coupling condition was reached. The deepest dip can be observed in the transmission curve presented in Figure 6e, and the Q-factor was 4.5 × 10^6^. In general, the Q factor should be higher for the critical coupling condition than that obtained in the undercoupling condition. Here, the slightly lower Q-factor measured in the critical coupling condition can be attributed to various imperfections in the fabrication process, which could influence the intrinsic Q of the microring itself beneath the coupling Si_3_N_4_ waveguide. When the distance was further reduced to 100 nm, a higher coupling efficiency was reached at the strong over-coupling, whilst the Q factor decreased to 2.6 × 10^6^, as illustrated in Figure 6c,f. The Q factor that we mentioned here is loaded Q factor.

## 4. Conclusions

To conclude, we have demonstrated efficient evanescent coupling between the crystalline LN microring resonator fabricated by PLACE and the Si_3_N_4_ waveguide fabricated by FIB with a vertical configuration. By controlling the distance between the waveguide and microresonator, nearly critical coupling condition has been achieved with ultra-high Q factors, i.e., the Q-factor of the fabricated LN microresonator was measured to be 1.49 × 10^7^. Furthermore, we demonstrated that the coupling efficiency can be continuously tuned upon demand by varying the thickness of the SiO_2_ cladding layer. We should point out that the coupling efficiency can also be tuned by changing the relative position between the waveguide and the microring resonator in the horizontal plane. The scheme proposed in this work is also beneficial for large-scale PIC integration, as multiple microresonators can be remotely connected on a single chip using the same waveguide and the coupling efficiency can be individually tuned, as reasoned above. Thus, the scheme provides a promising photonic integration solution widely adopted by a broad range of LNOI photonic applications, which range from micro/nano-nonlinear optics and optical interconnect to on-chip artificial intelligence demonstration, etc.

## References

[1] Boes A., Corcoran B., Chang L., Bowers J., Mitchell A. (2018). Status and potential of lithium niobate on insulator (LNOI) for photonic integrated circuits. Laser Photon. Rev..

[2] Diziain S., Geiss R., Zilk M., Schrempel F., Kley E.-B., Tünnermann A., Pertsch T. (2013). Mode analysis of photonic crystal L3 cavities in self-suspended lithium niobate membranes. Appl. Phys. Lett..

[3] Zhang M., Wang C., Cheng R., Shams-Ansari A., Lončar M. (2017). Monolithic ultra-high-Q lithium niobate microring resonator. Optica.

[4] Hu H., Yang J., Gui L., Sohler W. (2012). Lithium niobate-on-insulator (LNOI): Status and perspectives. Proc. SPIE.

[5] Geiss R., Saravi S., Sergeyev A., Diziain S., Setzpfandt F., Schrempel F., Grange R., Kley E.-B., Tünnermann A., Pertsch T. (2015). Fabrication of nanoscale lithium niobate waveguides for second-harmonic generation. Opt. Lett..

[6] Luo R., He Y., Liang H., Li M., Lin Q. (2019). Semi-nonlinear nanophotonic waveguides for highly efficient second-harmonic generation. Laser Photon. Rev..

[7] Krasnokutska I., Tambasco J.L.J., Li X.J., Peruzzo A. (2018). Ultra-low loss photonic circuits in lithium niobate on insulator. Opt. Express.

[8] Lu J., Surya J.B., Liu X., Xu Y., Tang H.X. (2019). Octave-spanning supercontinuum generation in nanoscale lithium niobate waveguides. Opt. Lett..

[9] He M., Xu M., Ren Y., Jian J., Ruan Z., Xu Y., Gao S., Sun S., Wen X., Zhou L. (2019). High-performance hybrid silicon and lithium niobate Mach–Zehnder modulators for 100 Gbit s−1 and beyond. Nat. Photon..

[10] Chen J.-Y., Ma Z.-H., Sua Y.M., Li Z., Tang C., Huang Y.-P. (2019). Ultra-efficient frequency conversion in quasi-phase-matched lithium niobate microrings. Optica.

[11] Luo R., He Y., Liang H., Li M., Lin Q. (2018). Highly tunable efficient second-harmonic generation in a lithium niobate nanophotonic waveguide. Optica.

[12] Wu R., Zhang J., Yao N., Fang W., Qiao L., Chai Z., Lin J., Cheng Y. (2018). Lithium niobate micro-disk resonators of quality factors above 107. Opt. Lett..

[13] Zhang J., Fang Z., Lin J., Zhou J., Wang M., Wu R., Gao R., Cheng Y. (2019). Fabrication of crystalline microresonators of high quality factors with a controllable wedge angle on lithium niobate on insulator. Nanomaterials.

[14] Wu R.B., Wang M., Xu J., Qi J., Chu W., Fang Z.W., Zhang J.H., Zhou J.X., Qiao L.L., Chai Z.F. (2018). Long low-loss-litium niobate on insulator waveguides with sub-nanometer surface roughness. Nanomaterials.

[15] Jiang W.C., Lin Q. (2016). Chip-scale cavity optomechanics in lithium niobate. Sci. Rep..

[16] Zhang M., Buscaino B., Wang C., Shams-Ansari A., Reimer C., Zhu R., Kahn J.M., Lončar M. (2019). Broadband electro-optic frequency comb generation in a lithium niobate microring resonator. Nature.

[17] Fang Z., Haque S., Farajollahi S., Luo H., Lin J., Wu R., Zhang J., Wang Z., Wang M., Cheng Y. (2020). Polygon coherent modes in a weakly perturbed whispering gallery microresonator for efficient second harmonic, optomechanical, and frequency comb generations. Phys. Rev. Lett..

[18] Wang L., Wang C., Wang J., Bo F., Zhang M., Gong Q., Lončar M., Xiao Y.-F. (2018). High-Q chaotic lithium niobate microdisk cavity. Opt. Lett..

[19] Wolf R., Breunig I., Zappe H., Buse K. (2018). Scattering-loss reduction of ridge waveguides by sidewall polishing Opt. Express.

[20] Pant M., Towsley D., Englund D., Guha S. (2019). Percolation thresholds for photonic quantum computing. Nat. Commun..

